# Brachial-ankle pulse wave velocity trajectories in a middle-aged population

**DOI:** 10.3389/fcvm.2023.1092525

**Published:** 2023-03-27

**Authors:** Xuan Deng, Yongjian Song, Xu Han, Xueyu Chen, Wenyi Yang, Shouling Wu, Yong Zhou

**Affiliations:** ^1^ Clinical Research Institute, Shanghai General Hospital, Shanghai Jiao Tong University School of Medicine, Shanghai, China; ^2^ Department of Cardiology, Zhangjiakou First Hospital, Zhangjiakou, China; ^3^ Department of Cardiology, Tianjin Medical University General Hospital, Tianjin, China; ^4^ Department of Biostatistics, School of Public Health, Cheeloo College of Medicine, Shandong University, Jinan, China; ^5^ Department of Cardiology, Shanghai General Hospital, Shanghai Jiao Tong University School of Medicine, Shanghai, China; ^6^ Department of Cardiology, Kailuan General Hospital, North China University of Science and Technology, Tangshan, China

**Keywords:** pulse wave velocity, arterial stiffness, arteriosclerosis, risk factor, trajectory

## Abstract

**Objective:**

The “trajectory” phenotype was observed in several cardiovascular risk factors with aging. We aim to identify multiple brachial-ankle Pulse Wave Velocity (baPWV) trajectory phenotypes and assess their determinants.

**Methods:**

Among 5,182 participants with baPWV measurements (2010–2016) at no less than three time points in Kailuan Study, we derived baPWV trajectory pattern using SAS Proc Traj program. We applied the lowest Bayesian information criterion to identify the best typing model, related the identified trajectory pattern to baseline and changes in characteristics.

**Results:**

Among 5.3 ± 1.7 years follow-up, four distinct baPWV trajectories were identified as low (1,961,37.8%), medium-low (1,846,35.6%), medium-high (1,024,19.8%), and high (351,6.8%) groups. In the stepwise models, mean arterial pressure and age were the main determinators of the trajectory patterns, with a Δpseudo-R^2^ of 0.335 and 0.164, respectively. With the low trajectory group as reference and multivariable adjustment, odd ratios of medium low, medium high and high associated with 1 mmHg increment of mean arterial pressure were 1.08(95%CI: 1.07–1.09), 1.13(1.12–1.14), and 1.16(1.15–1.18). The estimates for age were 1.08(1.07–1.10), 1.20(1.18–1.21) and 1.28(1.26–1.31). Additionally, baseline resting heart rate, low-density lipoprotein cholesterol, fasting blood glucose, hypersensitive C-reaction protein and uric acid, and changes in mean arterial pressure, resting heart rate, fasting blood glucose, and uric acid were positively associated with the trajectory, while BMI was negatively associated.

**Conclusions:**

The changes in baPWV overtime followed a “trajectory” pattern, mainly determined by mean arterial pressure and age.

## 1. Introduction

Aging has long been accepted as a non-modifiable risk factor and is highly associated with the progress of aging-related disease, such as arteriosclerosis (1). Arteriosclerosis is also a major hallmark of vascular aging and serves as an independent risk factor for cardiovascular and cerebrovascular diseases (CVD) (2–7). The progression of arteriosclerosis is affected by several factors, especially aging and hypertension (8, 9). However, previous studies have demonstrated that it may differ among individuals according to age, sex, and several traditional cardiovascular risk factors (8–12). Pulse wave velocity (PWV) has been used as a valuable index of arterial stiffness and as a surrogate marker for atherosclerosis (13). To date, some arteriosclerosis-related epidemiologic studies have only focused on brachial-ankle pulse wave velocity (baPWV) from one or a limited number of time-points, ignoring the dynamic changes in the baPWV index that occur over time. Hence, a life-course evaluation for arteriosclerotic progression is essential.

In some cohort studies, the “trajectory” phenotype was observed in several cardiovascular risk factors with aging (14–16), such as blood pressure, non-HDL, and body mass index (BMI), which suggests that the trajectory phenotypes of different risk factors might be a better index for CVD diagnosis and prognosis. However, the data on baPWV trajectory phenotypes in populations at high risk of cardiovascular diseases is limited. Therefore, this study aimed to identify multiple baPWV trajectory phenotypes and explore their risk factors in a Kailuan community-based cohort study with a 15-year follow-up.

## 2. Methods

### 2.1. Study design and population

The Kailuan Study (Registration Number: ChiCTR-TNC-11001489) was a community-based cohort study. Participants were recruited from Kailuan communities (Tangshan City, northern China) and follow-up information was obtained biennially from 2010 to 2016 (17). A total of 5,182 participants were involved in the final analyses after excluding those who did not complete a minimum of three assay repetitions of baPWV (*n* = 30,927); those with history of stroke, myocardial infarction, heart failure, or tumor (*n* = 100); and those with atrial fibrillation and an ankle-brachial index ≤0.9 (*n* = 160). This study was conducted according to the guidelines of the Helsinki Declaration and was approved by the Ethics Committee of Kailuan General Hospital. Signed informed consent was obtained from all participants.

### 2.2. Clinical characteristics

Clinical examination and questionnaire baseline data were collected by trained research staff according to standard operating procedures. The questionnaire included items on participant age, sex, smoking and drinking history, physical activity, current prescription medication, and medical history. Clinical examination included measurements of weight, height, waist circumference, resting heart rate, systolic blood pressure (SBP), and diastolic blood pressure (DBP). BMI was calculated as weight (kg)/height (m^2^) (18). Blood pressure and resting heart rate were measured *via* an Omron® electronic automated blood pressure monitor. Hypertension was defined as a SBP ≥140 mmHg or a DBP ≥90 mmHg, a history of hypertension, or treatment with antihypertensive medications (19). Diabetes mellitus was a self-reported diagnosis, a fasting blood glucose (FBG) level of ≥7.0 mmol/L (126 mg/dl), or use of antidiabetic agents (20).

### 2.3. Laboratory tests

Blood samples of all participants were collected by venipuncture. FBG, low-density lipoprotein cholesterol (LDL-C), serum uric acid, and high-sensitivity C-reactive protein (Hs-CRP) were determined by the Friedewald formula (11). The estimated glomerular filtration rate (eGFR) was calculated according to the Chronic Kidney Disease Epidemiology Collaboration formula (21).

### 2.4. Assessment of baPWV

BaPWV was measured in some participants from 2010 (the third follow-up), and the baPWV of the study population was repeatedly assessed during each follow-up. It was measured by an Omron Colin BP-203RPE III device (Omron Health Care, China). Before the measurement, the participants were instructed not to smoke and had to rest for at least 5 min. The temperature of the examination room was maintained at 22–25°C. The participant was placed in a supine position, and cuffs were wrapped on both arms and ankles. The heart sound collection device was placed in the pre-heart area. The electrocardiogram (ECG) collection device was clamped on the left and right wrists and connected to the plethysmography sensor and the oscillation pressure sensor. We repeated the measurement twice for each participant, and recorded the second data as the final result; we used the larger value of baPWV on the left and right sides for analysis. We categorized the levels of baPWV into three grades: low (<14.0 m/s), intermediate (14.0–17.9 m/s), and high (≥18.0 m/s) (22).

### 2.5. Statistical analysis

The trajectories of baPWV were identified using PROC TRAJ in SAS® v.9.4 (SAS Institute Inc, Cary, NC, United States) (23). The best typing model was selected based on the lowest Bayesian information criterion. After the typing was determined, the highest power of each trajectory curve was determined according to the probability (>70%) that the observed object meets the trajectory curve. The Kolmogorov-Smirnov test was used to test for a normal distribution of continuous variables. Data with a normal distribution were expressed as mean ± standard deviation and analyzed by one-way analysis of variance. Categorical data were presented as frequencies and percentages (%), and differences between types were evaluated by *χ*^2^ test.

Multivariate regression analysis was used to evaluate the association of baPWV trajectory with baseline data. We adjusted covariates including age, sex, mean arterial pressure (MAP, computed as 1/3 SBP + 2/3 DBP), resting heart rate, BMI, waist circumference, FBG, LDL-C, Hs-CRP, uric acid, and eGFR. Multiple logistic regression analysis was used to explore factors associated with baPWV longitudinal trajectory. The forward stepwise method was used for multiple logistic regression (enter probability: 0.05, remove probability: 0.10). In addition, we performed a multiple analysis with adjustment of the following variables: Δ MAP, Δ resting heart rate, Δ BMI, Δ waist circumference, Δ FBG, ΔLDL-C, ΔHs-CRP, Δ uric acid, and Δ eGFR to assess their effect on the baPWV longitudinal trajectory during the follow-up. A likelihood ratio test was used to assess the significance of the model fit. The effectiveness of the model was estimated with the Nagelkerke pseudo-R^2^ statistic (a measure of explained variation in the model). Sensitivity analysis was performed based on baseline antihypertensive drugs, new-onset hypertension, diabetes, and participants with cardiovascular disease during the follow-up period. A two-sided *p* < 0.05 was considered statistically significant. Statistical analyses were performed using SAS® v.9.4 (SAS Institute Inc).

## 3. Results

### 3.1. Baseline and follow-up characteristics of participants

The study included totally 5,182 participants, of whom 2,445 (47.18%) were male. The average age was 46.87 ± 9.57 years at baseline. The median follow-up year is 5.66 years (Interquartile range, 4.12–6.52 years). Supplementary Table S1 summarizes the baseline and follow-up characteristics of the participants. Compared with baseline levels, the following variables significantly increased (*p* < 0.001): MAP, resting heart rate, LDL-C, BMI, waist circumference, FBG, and uric acid. The prevalence of hypertension and diabetes also increased. More participants during follow-up received antihypertensive medications, anti-diabetic medications, or lipid-lowering medications. In contrast, the proportion of physical exercisers, smokers, and alcohol drinkers decreased. All the above trends were statistically significant (*p* < 0.001). A comparison between the baseline characteristics of the included participants and the general population who only underwent a single baPWV measurement is shown in Supplementary Table S2. Compared with the general population, the participants tended to have lower levels of MAP, resting heart rate, waist circumference, BMI, LDL-C, FBG, Hs-CRP, and uric acid, with a relatively elevated eGFR level. In addition, there was a lower prevalence of high/low-density lipoproteinemia and diabetes in the study population, and relatively fewer smokers, but more alcohol drinkers.

### 3.2. BaPWV by different trajectory groups

**Figure 1:**
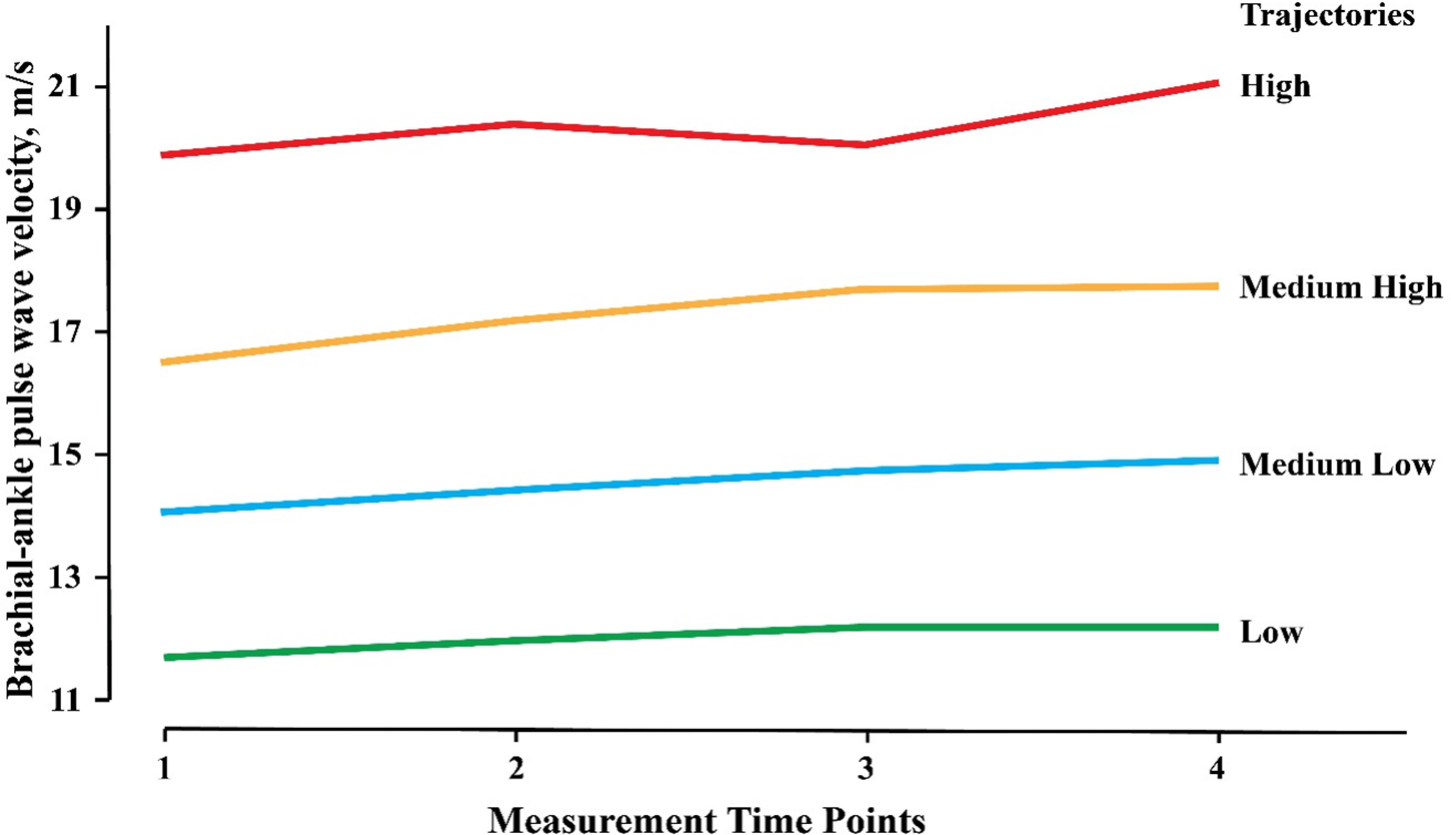
Trajectories of baPWV among 5182 participants. The baPWV Trajectories derived from 17,331 consecutive baPWV measurements in 5,182 participants enrolled in the Kailuan Study and had at least three times of measurements. baPWV, brachial-ankle pulse wave velocity.

A total of 17,331 baPWV measurements were performed among 5,182 participants, with a mean of 3.34 ± 0.48 times for each. The annual growth rate of baPWV was 0.14 ± 0.40 m/s/y. Four baPWV trajectories were identified, including a low group, medium-low group, medium-high group, and high group. In the low group (1,961 cases, 37.84%), the mean baPWV increased from 11.70 to 12.24 m/s, with an increase of 0.54 m/s and an annual growth rate of 0.10 m/s/y. In the medium-low group (1,846 cases, 35.62%), the mean baPWV increased from 14.07 to 14.94 m/s, with an increase of 0.87 m/s and an annual growth rate of 0.16 m/s/y. In the medium-high group (1,024 cases, 19.76%), the mean baPWV increased from 16.53 to 17.75 m/s, with an increase of 1.22 m/s and an annual growth rate of 0.21 m/s/y. In the high group (351 cases, 6.77%), the mean value of baPWV increased from 19.90 to 21.09 m/s, with an increase of 1.19 m/s and an annual growth rate of 0.15 m/s/y. The probability of each type (low group, medium-low group, medium-high group, and high group) meeting the trajectory curve was 90.72%, 84.86%, 87.33%, and 91.93%, respectively (Figure 1 and Table 1). The trajectories of males and females were consistent with that of the total participants. The initial baPWV of each trajectory type in females was lower than that in males, while there was no difference observed in the baPWV annual growth rate (0.14 m/s/y) (Supplementary Figure S1 and Tables S3, S4).

**Table 1:** Baseline characteristics by baPWV trajectory groups.

Characteristics	Low (*n* = 1,961)	Medium-low (*n* = 1,846)	Medium-high (*n* = 1,024)	High (*n* = 351)
**Number (%) of participants**				
Women	1505 (76.7)	784 (42.5)	335 (32.7)	113 (32.2)
Current smoking	220 (11.2)	560 (30.3)	366 (35.7)	123 (35.0)
Drinking alcohol	54 (2.8)	146 (7.9)	128 (12.5)	52 (14.8)
Physical exercises	170 (24.1)	249 (13.5)	207 (20.2)	79 (22.5)
Hypertension	210 (10.7)	811 (43.9)	731 (71.4)	310 (88.3)
On antihypertensive treatment	54 (2.81)	242 (13.3)	314 (31.0)	163 (46.8)
Diabetes mellitus	40 (2.0)	147 (8.0)	174 (17.0)	104 (29.6)
On antidiabetic treatment	10 (0.5)	49 (2.7)	81 (8.0)	55 (15.7)
On Lipid-lowering drugs	11 (0.9)	29 (2.1)	30 (3.5)	9 (2.9)
**Mean characteristic**				
Age (year)	41.1 ± 6.9	46.3 ± 7.5	52.4 ± 9.8	57.8 ± 11.3
BMI (kg/m^2^)	23.7 ± 3.2	25.0 ± 3.3	25.6 ± 3.4	25.4 ± 3.2
Waist circumference (cm)	79.7 ± 9.2	85.0 ± 9.4	88.4 ± 9.4	88.6 ± 9.4
Heart beat (beats/min)	70.4 ± 10.2	71.9 ± 9.5	73.5 ± 10.6	75.1 ± 10.7
Systolic BP (mm Hg)	114.3 ± 13.8	125.9 ± 14.8	135.8 ± 16.5	142.6 ± 16.1
Diatolic BP (mm Hg)	75.3 ± 8.8	82.8 ± 9.8	86.7 ± 10.6	87.6 ± 10.5
MAP (mm Hg)	88.3 ± 9.9	97.2 ± 10.7	103.1 ± 11.6	105.9 ± 10.6
LDL-cholesterol (mmol/L)	2.32 ± 0.74	2.58 ± 0.76	2.69 ± 0.93	2.69 ± 0.81
FBG (mmol/L)	5.03 ± 0.70	5.44 ± 1.52	5.80 ± 1.77	6.30 ± 2.22
Uric acid (µmol/L)	263.9 ± 73.1	288.2 ± 90.2	308.2 ± 96.8	307.6 ± 93.8
eGFR [ml/(min*1.73 m^2^)]	105.4±18.9	99.7±21.2	96.9±19.3	93.3 ± 22.4
Hs-CRP (mg/L)	0.80 (0.40, 1.60)	1.04 (0.45, 2.10)	1.10 (0.60, 2.70)	1.28 (0.70, 2.84)

BMI, body mass index; BP, blood pressure; eGFR, estimated glomerular filtration rate; FBG, fasting blood glucose; Hs-CRP, hypersensitive C-reactive protein; LDL, low-density lipoprotein; MAP, mean arterial pressure. Reported values are number of participants (%), arithmetic means (±SD) or geometric means (interquartile range). Hypertension was a office blood pressure of ≥140 mmHg systolic or ≥90 mm Hg diastolic or use of antihypertensive drugs. Diabetes mellitus was a self-reported diagnosis, a fasting blood glucose level of ≥7.0 mmol/L (126 mg/dl), or use of antidiabetic agents. The estimated glomerular filtration rate (eGFR) was calculated according to the Chronic Kidney Disease Epidemiology Collaboration formula. The baPWV trajectory groups were derived from 17,331 consecutive baPWV measurements in 5,182 participants. *P*-values for trend difference across the four groups are all ≤0.001.

### 3.3. Baseline and follow-up characteristics among baPWV trajectories

A comparison of the baseline characteristics among different baPWV trajectory groups is shown in Table 2. With an increasing baPWV longitudinal trajectory level, the following covariates showed an upward trend: age; male proportion; SBP; DBP; MAP; resting heart rate; BMI; waist circumference; LDL-C; FBG; Hs-CRP; uric acid; proportion of smokers, alcohol drinkers, and physical exercisers; and participants with history of hypertension and diabetes, and antihypertensive, anti-diabetic, and lipid-lowering medications. In contrast, the level of eGFR decreased. All the above trends were statistically significant (*p* < 0.001). Changes in the main independent variable parameters among different trajectory groups during follow-up are presented in Table 3. Δ FBG and Δ uric acid significantly increased (*p* < 0.01), and the percentage of participants with new-onset hypertension, diabetes, and cardiovascular disease also increased (*p* < 0.001). However, levels of Δ BMI, Δ waist circumference, Δ LDL-C, and Δ eGFR significantly decreased during follow-up (*p* < 0.001).

**Table 2:** Consecutive baPWV measurements across four trajectory groups.

BaPWV	Low (*n* = 1,961)	Medium-low (*n* = 1,846)	Medium-high (*n* = 1,024)	High (*n* = 351)
BaPWV_1_	11.70 ± 1.11	14.07 ± 1.30	16.53 ± 1.77	19.90 ± 2.22
N_1_ (5,176)	1961	1846	1021	348
≤14 m/s	1,921 (98.0)	970 (52.6)	53 (5.2)	0 (0)
14–18 m/s	40 (2.0)	860 (46.6)	770 (75.4)	70 (20.1)
>18 m/s	0 (0)	16 (0.9)	198 (19.4)	278 (79.9)
BaPWV_2_	11.98 ± 1.17	14.47 ± 1.41	17.19 ± 1.83	20.42 ± 2.17
N_2_ (5,153)	1,960	1,845	1,009	343
≤14 m/s	1,897 (96.8)	741 (40.3)	23 (2.3)	0 (0)
14–18 m/s	63 (3.2)	1,080 (58.7)	690 (68.4)	49 (2.6)
>18 m/s	0 (0)	20 (1.1)	296 (29.3)	294 (85.7)
BaPWV_3_	12.19 ± 1.19	14.74 ± 1.44	17.72 ± 1.96	20.91 ± 2.21
N_3_ (5,161)	1,960	1,845	1,017	340
≤14 m/s	1,871 (95.5)	559 (30.3)	21 (2.1)	2 (0.6)
14–18 m/s	87 (4.4)	1,252 (67.3)	572 (56.2)	35 (10.3)
>18 m/s	1 (0.1)	34 (1.8)	342 (41.7)	303 (89.1)
BaPWV_4_	12.24 ± 1.32	14.94 ± 1.43	17.75 ± 1.99	21.09 ± 2.19
N4 (1841)	834	615	288	104
≤14 m/s	774 (92.8)	163 (26.5)	7 (2.4)	1 (1.0)
14–18 m/s	60 (7.2)	436 (70.9)	170 (59.0)	8 (7.7)
>18 m/s	0 (0)	16 (2.60)	111 (38.5)	95 (91.3)
Annual Δ baPWV (m/s per year)	0.10 ± 0.30	0.16 ± 0.37	0.21 ± 0.54	0.15 ± 0.72

BaPWV, brachial-ankle pulse wave velocity. BaPWV1-4 are the first to fourth measurements. Annual ΔbaPWV was the difference of baPWV4 and baPWV1 divided by duration of follow-up in years. Reported values are arithmetic means (±SD) for the four baPWV measurements and Annual ΔbaPWV, number of avaible measurement during each follow-up or the number of participants (%) across three categories based on the thresholds of ≤14, 14–18 and >18 m/s within each follow-up. *P*-values for trend difference across four trajectory groups are all <0.001.

**Table 3:** Changes in variables across baPWV trajectory groups.

Variables	Low (*n* = 1,961)	Medium-low (*n* = 1,846)	Medium-high (*n* = 1,024)	High (*n* = 351)	*p* for trend
**Number (%) of participants**					
New-onset hypertension	123 (6.3)	272 (14.7)	150 (14.7)	25 (7.1)	<0.001
New-onset diabetes	19 (1.0)	86 (4.7)	74 (7.2)	30 (8.6)	<0.001
New-onset CVD	4 (0.2)	19 (1.0)	26 (2.5)	21 (5.9)	<0.001
**Mean characteristics**					
Δ MAP (mmHg)	1.81 ± 9.6	1.60 ± 11.2	2.60 ± 12.9	1.87 ± 13.9	0.19
Δ Resting heart rate (bpm)	1.40 ± 16.8	1.71 ± 14.5	2.60 ± 17.2	2.02 ± 12.5	0.29
Δ BMI (kg/m^2^)	0.56 ± 3.12	0.19 ± 2.67	0.01 ± 3.01	−0.11 ± 2.30	<0.001
Δ Waist Circumference (cm)	2.03 ± 8.85	1.12 ± 9.10	0.30 ± 8.82	0.28 ± 8.66	<0.001
Δ LDL-cholesterol (mmol/L)	0.48 ± 1.06	0.37 ± 1.08	0.28 ± 1.30	0.28 ± 0.89	<0.001
Δ FBG (mmol/L)	0.16 ± 1.39	0.39 ± 2.92	0.59 ± 2.76	0.74 ± 3.93	<0.001
Δ Uric acid (µmol/L)	20.4 ± 66.2	28.9 ± 87.5	27.6 ± 92.7	35.7 ± 104.1	0.002
Δ Hs-CRP (mg/L)	−0.03 (−0.72, 0.60)	0 (−0.86, 0.61)	0 (−0.96, 1.20)	−0.04 (−1.15, 1.00)	0.64
Δ eGFR [ml/(min*1.73 m^2^)]	−3.48 (−10.4, 5.25)	−3.45 (−10.4, 7.36)	−3.54 (−11.8, 5.89)	−5.00 (−13.8, 2.62)	0.020

BMI, body mass index; CVD, cardiovascular disease; eGFR, estimated glomerular filtration rate; FBG, fasting blood glucose; Hs-CRP, hypersensitive C-reactive protein; LDL, low-density lipoprotein; MAPvmean arterial pressure. Δ was the difference of last follow-up data and baseline data.Reported values are number of participants (%), arithmetic means (±SD) or geometric means (interquartile range). Hypertension was a office blood pressure of ≥140 mmHg systolic or ≥90 mm Hg diastolic or use of antihypertensive drugs. Diabetes mellitus was a self-reported diagnosis, a fasting blood glucose level of ≥7.0 mmol/L (126 mg/dl), or use of antidiabetic agents. The estimated glomerular filtration rate (eGFR) was calculated according to the Chronic Kidney Disease Epidemiology Collaboration formula.

### 3.4. Factors relevant to baPWV longitudinal trajectory

We also explored the factors relevant to the baPWV longitudinal trajectory. Compared to the low trajectory group, older participants, males, alcohol drinkers, and those with higher levels of MAP, resting heart rate, FBG, LDL-C, Hs-CRP, and uric acid tended to have a higher risk of inclusion in the high trajectory groups. We further investigated the effect of changes in the above-mentioned variables on the baPWV longitudinal trajectory during the follow-up. After fully adjusting, baseline MAP, resting heart rate, FBG, and uric acid were positively associated with the baPWV trajectory. In addition, the participants with increased ΔMAP, Δ resting heart rate, ΔFBG, and Δ uric acid had an increased risk of inclusion into the higher trajectory group. The fitting degree of the model increased by 5.1%–59.1% after adjustment (Table 4).

**Table 4:** Association of baseline measurements and changes in variables with baPWV trajectories.

		Low	Medium-low	*P*	Medium-high	*P*	High	*P*
		(*n* = 1,961)	(*n* = 1,846)		(*n* = 1,024)		(*n* = 351)	
Model 1	Age	Ref.	1.08 (1.07–1.10)	<0.001	1.20 (1.18–1.21)	<0.001	1.28 (1.26–1.31)	<0.001
	Gender	Ref.	2.02 (1.68–2.42)	<0.001	2.22 (1.76–2.81)	<0.001	2.28 (1.73–3.39)	<0.001
	MAP	Ref.	1.08 (1.07–1.09)	<0.001	1.13 (1.12–1.14)	<0.001	1.16 (1.15–1.18)	<0.001
	Resting heart rate	Ref.	1.02 (1.01–1.03)	<0.001	1.04 (1.03–1.05)	<0.001	1.07 (1.05–1.08)	<0.001
	FBG	Ref.	1.20 (1.10–1.31)	<0.001	1.33 (1.21–1.47)	<0.001	1.52 (1.37–1.69)	<0.001
	LDL- cholesterol	Ref.	1.18 (1.06–1.31)	0.001	1.21 (1.05–1.39)	<0.001	1.15 (0.94–1.40)	0.070
	Hs-CRP	Ref.	1.02 (1.00–1.05)	0.012	1.05 (1.05–1.08)	0.001	1.02 (0.98–1.07)	0.112
	Uric acid	Ref.	1.001 (1.000–1.003)	0.124	1.003 (1.001–1.004)	0.001	1.002 (1.001–1.004)	0.023
	Drinking alcohol	Ref.	0.91 (0.64–1.31)	0.453	1.26 (0.85–1.88)	0.498	1.79 (1.09–2.94)	0.007
Model 2	Age	Ref.	1.08 (1.07–1.09)	<0.001	1.20 (1.18–1.22)	<0.001	1.29 (1.27–1.32)	<0.001
	Gender	Ref.	1.80 (1.49–2.17)	<0.001	1.83 (1.42–2.35)	<0.001	1.92 (1.34–2.74)	<0.001
	MAP	Ref.	1.11 (1.10–1.12)	<0.001	1.20 (1.18–1.22)	<0.001	1.25 (1.23–1.27)	<0.001
	Resting heart rate	Ref.	1.03 (1.02–1.04)	<0.001	1.06 (1.05–1.07)	<0.001	1.08 (1.06–1.09)	<0.001
	BMI	Ref.	0.96 (0.93–0.99)	<0.001	0.95 (0.91–1.00)	0.067	0.90 (0.85–0.96)	<0.001
	FBG	Ref.	1.24 (1.13–1.37)	<0.001	1.40 (1.26–1.56)	<0.001	1.64 (1.46–1.85)	<0.001
	LDL- cholesterol	Ref.	1.27 (1.12–1.44)	<0.001	1.23 (1.04–1.46)	0.006	1.11 (0.88–1.41)	0.195
	Uric acid	Ref.	1.002 (1.000–1.003)	0.059	1.003 (1.002–1.005)	0.002	1.004 (1.001–1.006)	0.003
	Drinking alcohol	Ref.	0.83 (0.57–1.19)	0.386	1.12 (0.75–1.69)	0.505	1.66 (0.99–2.76)	0.056
	Δ MAP	Ref.	1.06 (1.05–1.07)	<0.001	1.11 (1.09–1.12)	<0.001	1.13 (1.11–1.14)	<0.001
	Δ Resting heart rate	Ref.	1.01 (1.00–1.02)	0.051	1.02 (1.01–1.03)	<0.001	1.01 (1.00–1.02)	0.001
	Δ FBG	Ref.	1.14 (1.04–1.24)	0.001	1.21 (1.09–1.33)	<0.001	1.30 (1.16–1.45)	<0.001
	Δ LDL-cholesterol	Ref.	1.09 (0.97–1.22)	0.353	0.92 (0.79–1.06)	0.105	0.82 (0.67–1.01)	0.287
	Δ Uric acid	Ref.	1.002 (1.000–1.003)	0.090	1.003 (1.001–1.004)	0.005	1.005 (1.003–1.007)	<0.001

BMI, body mass index; FBG, fasting blood glucose; Hs-CRP, hypersensitive C-reactive protein; LDL, low-density lipoprotein; MAP, mean arterial pressure. Δ was the difference of last follow-up data and baseline data. Gender (female) is the reference group. Reported values are odds ratios with 95% confidence intervals which indicated each change of one unit increased the risk of inclusion in the higher trajectory group. Model 1: longitudinal trajectory of baPWV is the independent variable (low group is the control group). Baseline data obtained at the first baPWV measurement is the dependent variable. Multiple logistic regression analysis results show that the value of the Nagelkerke pseudo-R^2^ of the model is 0.540. Model 2: based on Model 1, Δ MAP, Δ Rest heart rate, Δ FBG, Δ LDL- cholesterol, and Δ Uric acid are additionally adjusted, and the value of the Nagelkerke pseudo-R^2^ of the model is 0.59.

### 3.5. Factors contributing to baPWV trajectory

The 54% variation of the baPWV trajectory was interpreted by baseline data: 33.5% by MAP, 16.4% by age, 1.3% by sex, and 1.3% by resting heart rate. The rest of the variation was interpreted by a combination of FBG, uric acid, Hs-CRP, LDL-C, and drinking. After adding the adjusted value of the independent variable, the interpretation degree of the variation of the baPWV trajectory increased by 5.1%, of which 49.9% was still interpreted by MAP (33.5%) and age (16.4%), while the rest was interpreted by ΔMAP (5.4%), resting heart rate (1.2%), and other variables (Supplementary Table S5). In addition, the effect relationship between age and baPWV was found by restricted cubic spline plot (RCS). A positive correlation exists between age and baPWV, and the baPWV value gradually increases with age (Supplementary Figure S3).

### 3.5. Sensitivity analysis

A sensitivity analysis was conducted on baseline antihypertensive medication, new-onset hypertension, new-onset diabetes, and new-onset CVD population. We found that advanced age; male sex; high levels of MAP, resting heart rate, FBG, LDL-C, and uric acid; low BMI; and increased MAP, resting heart rate, FBG, and uric acid remained risk factors for high baPWV level (Supplementary Table S6).

## 4. Discussion

This longitudinal study indicated that there were four trajectories of arteriosclerosis progression in middle-aged participants: low, medium-low, medium-high, high trajectories. These trajectories had good intra-type homogeneity and inter-type heterogeneity. They were independent and did not overlap. There was a significant difference in the baPWV baselines and growth rates of trajectories. The trajectory curve fitted well. The majority of participants with a healthy condition (92.8%) belonged to the low group and those with arteriosclerosis (79.89%) were mainly in the high group. The number of participants in the high group accounted for about 7.0%. The probability of meeting the trajectory in the medium-low type was the lowest (85.0%). The trajectories determined in our study met the trajectory standard proposed by Nagin et al. (23). Hence, the trajectory was relatively objective and might not be caused by random errors or confounding factors.

The study showed that advanced age and high MAP were the main risk factors for high baPWV. Our findings are consistent with previous studies that have suggested an association between advanced age and arteriosclerosis in the general population, diabetic population, and hypertensive population (2, 24–26). Considering the complex association between blood pressure and arteriosclerosis, we used MAP as an independent variable to observe its effect on the trajectories of arteriosclerosis. Our findings are consistent with previous studies that have indicated an association between baseline and changes in MAP, and the progression of arteriosclerosis. Furthermore, antihypertensive treatment could only slow down the progression of arteriosclerosis caused by advanced age (27). In our previous study, we also found that the hypertension caused by aging could be mediated by arteriosclerosis (24). The high MAP might be the result of the high trajectory of arteriosclerosis. Therefore, we suggested that the relationship between hypertension and arteriosclerosis is bidirectional, and arteriosclerosis plays a leading role. In addition, We also found that baPWV increases with age, especially between 45 and 55 years old, which means that the change of baPWV will accelerate after 45 years old. Our result is comparable to a cross-sectional study (28).

Compared with age and MAP, factors including baseline resting heart rate, FBG, L-DLC, hs-CRP, and uric acid had less effect on the trajectories of arteriosclerosis. Our findings are consistent with previous studies. Some studies have found that resting heart rate or hs-CRP were risk factors for arteriosclerosis in males (29–31). Nevertheless, the effects of glucose and lipid metabolic abnormalities on the progression of arteriosclerosis are inconsistent (32–35). Apart from different study populations, these inconsistencies also might be caused by the weak effect of these factors on arteriosclerosis. Changes in risk factors during follow-up also influenced the trajectories. An increase in MAP, resting heart rate, FBG, or uric acid during follow-up increased the risk of inclusion into the high group, while this increase of risk was less than that caused by baseline data. Baseline data represents the exposure of risk factors accumulated in the decades before the measurement of baPWV. Therefore, it has a large impact on the trajectories. While the increase in MAP, resting heart rate, FBG, and uric acid during follow-up could only represent the exposure level of risk factors during follow-up. This explains why it had little effect on the trajectories. Although we included traditional cardiovascular risk factors and possible influencing factors in the model, the whole model could only explain a portion (59.1%) of the trajectories. We speculated that the unexplained part is determined by individual genetic factors (36).

We also explored the baPWV trajectory in different sexes, and the trajectories are similar to the total population. Compared with males, the initial bapwv of females in each trajectory group is slightly smaller. However, we did not observe the significant difference in the annual growth rate between males and females. In other studies, there is also no significant difference of baPWV was observed between the sexes (37). However, the gender difference some studies have proposed the differences in relative young population aged from 4 to 29 years (38). We speculated this inconsistency might be caused the different age range of the study population.

Our study has some limitations. First, baPWV is currently recognized as a good indicator of arterial stiffness, but it is not the gold standard for the diagnosis of arteriosclerosis. Second, although we incorporated traditional cardiovascular risk factors and other possible risk factors in the model, we did not consider the influence of genetic factors on baPWV. In addition, consideration of the adjusted blood pressure effect on baPWV levels was missing in this study. In our study, the observation about the trajectory change of baPWV and the intervention of baPWV level is missing, which might be more valuable to predict the future atherosclerotic disease. Finally, the trajectories of baPWV change in a middle-aged population in this study are limited to 5 years.

## Conclusions

To our knowledge, the current study is the first to demonstrate the dynamic changes in the baPWV. We observed the change of baPWV showed a “trajectory” pattern in the middle-aged population, and we also explored the factors associated with baPWV trajectory and identified that age and MAP were the two main determinants of baPWV trajectory.

## Data Availability

The datasets used and/or analyzed during the current study are available from the corresponding author on reasonable request.
