# Supplementary material for: Brachial-ankle pulse wave velocity trajectories in a middle-aged population
Source: Front Cardiovasc Med. 2023 Mar 27;10:1092525. doi: 10.3389/fcvm.2023.1092525 (PMC10083284; doi:10.3389/fcvm.2023.1092525)
Supplement: Supplementary file 1 [file Table1.docx]

Supplementary Material

# Supplementary Figures and Tables

**Table of Contents**

**Supplementary Figure 1** The baPWV trajectories by gender Page 2

**Supplementary Figure 2** The effect relationship between age and baPWV Page 3

**Supplementary Table 1** Characteristics of participants at baseline and the last follow-up Page 4

**Supplementary Table 2** Comparison of included/overall participants among the Kailuan population

Page 5

**Supplementary Table 3** BaPWV by different trajectory groups in males Page 6

**Supplementary Table 4** BaPWV by different trajectory groups in females Page 7

**Supplementary Table 5** Fitting degree of the main risk factors for the trajectory models Page 8

**Supplementary Table 6** Sensitive analyses by excluding participants with antihypertensive treatment or incidence of hypertension, diabetes mellitus, or cardiovascular disease. Page 10

**A.**


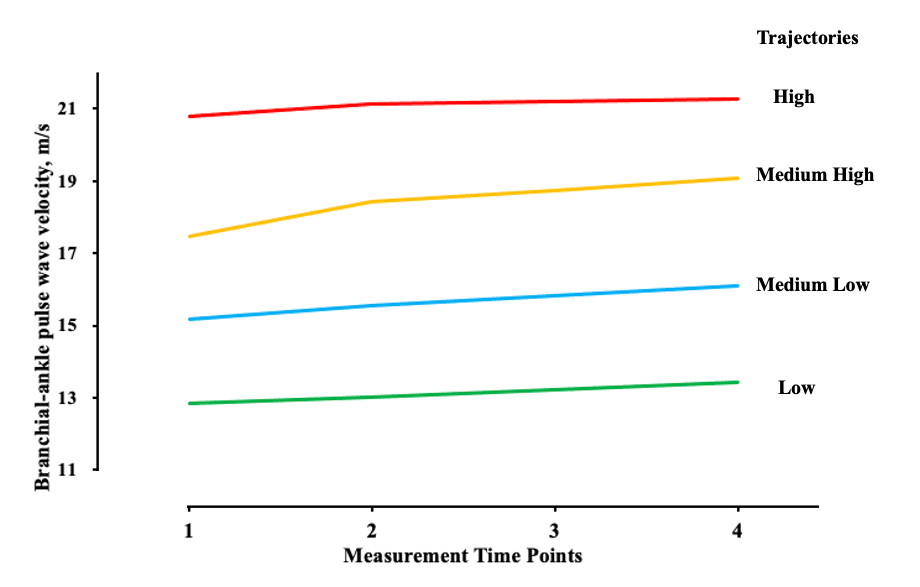


**B.**


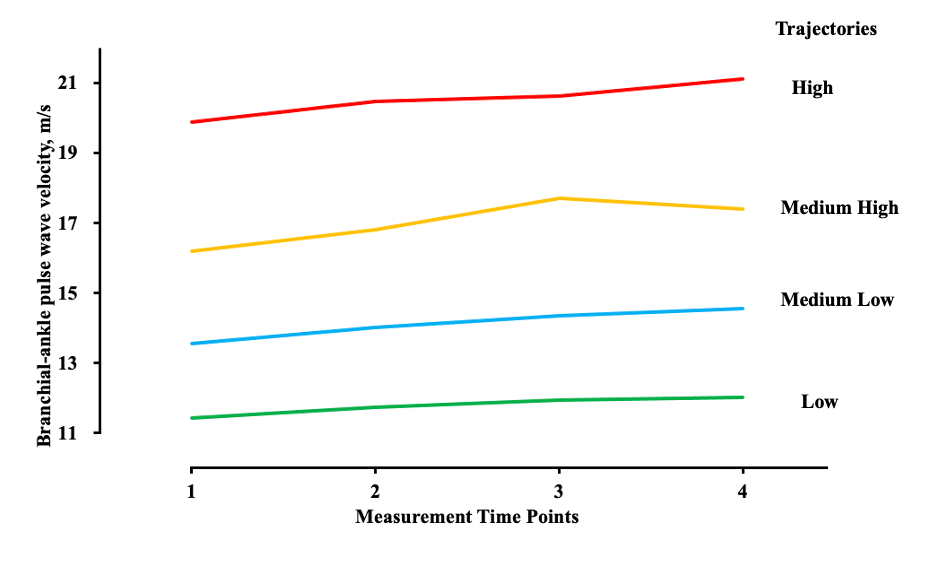


**Supplementary Figure 1.** The baPWV trajectories by gender. (A) The baPWV trajectories in male participants. (B) The baPWV trajectories in female participants. The trajectory classification derived for the 5182 participants who underwent at least three baPWV assessments in the Kailuan Study. baPWV = brachial-ankle pulse wave velocity


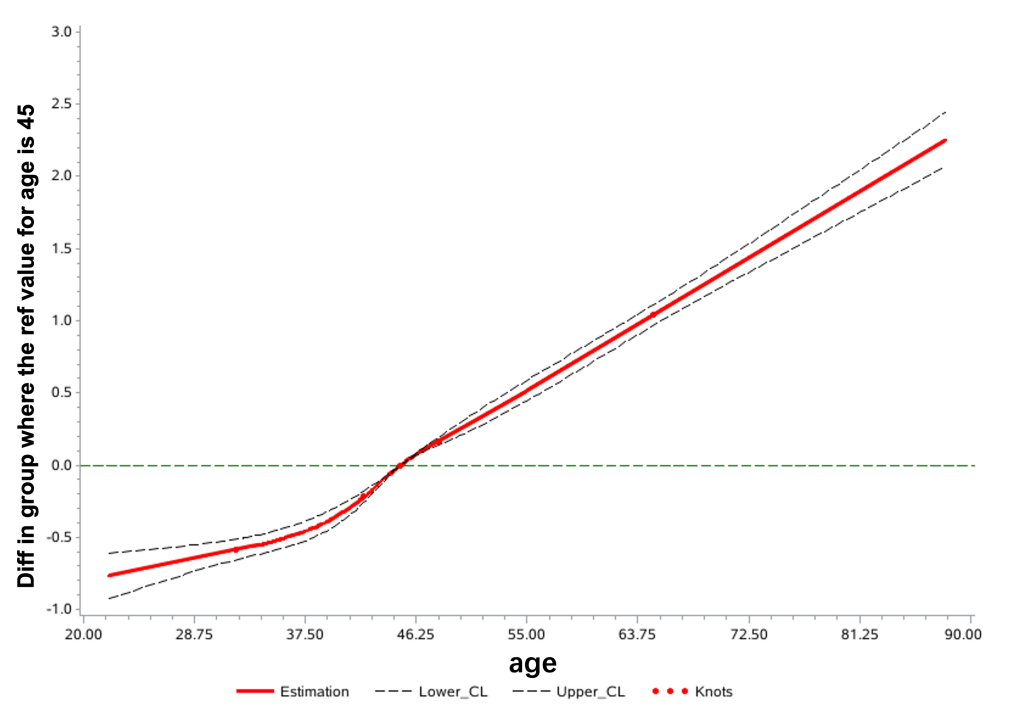


**Supplementary Figure 2.** The effect relationship between age and baPWV

**Supplementary Table 1.** Characteristics of participants at baseline and the last follow-up.

| **Characteristics** | **Baseline data**  **(n=5,182)** | **Follow-up data**  **(n=5,182)** | **p value** |
| --- | --- | --- | --- |
| Number (%) of participants |  |  |  |
| Males | 2,445 (47.3) | 2,445 (47.2) |  |
| Current smoking | 1,269 (24.5) | 819 (15.8) | <0.001 |
| Drinking alcohol | 380 (7.3) | 124 (2.4) | <0.001 |
| Physical exercises | 705 (13.6) | 212 (4.1) | <0.001 |
| Hypertension | 2,062 (39.8) | 2,632 (50.8) | <0.001 |
| On antihypertensive treatment | 773 (15.1) | 982 (20.3) | <0.001 |
| Diabetes mellitus | 465 (9.0) | 674 (13.0) | <0.001 |
| On antidiabetic treatment | 195 (3.8) | 218 (11.7) | <0.001 |
| On Lipid-lowering drugs | 79 (2.1) | 94 (6.1) | <0.001 |
| Mean characteristics |  |  |  |
| Age (year) | 46.9±9.6 | 50.4±10.2 | <0.001 |
| MAP (mm Hg) | 94.9±12.7 | 97.0±13.0 | <0.001 |
| Resting heart rate (bpm) | 72.3±10.8 | 74.0±16.8 | <0.001 |
| Waist circumference (cm) | 83.9±10.0 | 85.2±9.5 | <0.001 |
| BMI (kg/m2) | 24.2±2.8 | 24.4±2.8 | <0.001 |
| LDL- cholesterol (mmol/L) | 2.5±0.7 | 2.9±0.8 | <0.001 |
| FBG (mmol/L) | 5.4±1.4 | 5.7±1.6 | <0.001 |
| Uric acid (µmol/L) | 306.9±77.8 | 323.0±78.8 | <0.001 |
| eGFR (mL/min·1.73m^2^) | 100.2±19.2 | 100.5±16.1 | 0.392 |
| Hs-CRP (mg/L) | 1.08 (0.50,1.97) | 1.06 (0.49,1.94) | 0.352 |

BMI = body mass index; eGFR = estimated glomerular filtration rate; FBG = fasting blood glucose; Hs-CRP = hypersensitive C-reactive protein; LDL- cholesterol = low-density lipoprotein cholesterol; MAP = mean arterial pressure; RHR = resting heart rate; Reported values are number of participants (%), arithmetic means (±SD) or geometric means (interquartile range). Hypertension was an office blood pressure of ≥140 mmHg systolic or ≥90 mm Hg diastolic or use of antihypertensive drugs. Diabetes mellitus was a self-reported diagnosis, a fasting glucose level of ≥7.0mmol/L (126 mg/dL), or use of antidiabetic agents. The estimated glomerular filtration rate (eGFR) was calculated according to the Chronic Kidney Disease Epidemiology Collaboration formula.

**Supplementary Table 2.** Comparison of included/overall participants among the Kailuan population.

| **Characteristics** | **Included participants**  **(n=5,182)** | **Overall participants**  **(n=36,352)** | **p value** |
| --- | --- | --- | --- |
| Number (%) of participants |  |  |  |
| Males | 2,445 (47.2) | 3,573 (72.0) | <0.001 |
| Current smoking | 1,269 (24.5) | 14,773 (29.8) | <0.001 |
| Drinking alcohol | 380 (7.3) | 2,244 (4.5) | <0.001 |
| Physical exercises | 705 (13.6) | 3,917 (7.9) | <0.001 |
| Hypertension | 2,062 (39.8) | 19,707 (39.7) | 0.886 |
| On antihypertensive treatment | 773 (15.1) | 6,259 (13.7) | 0.0039 |
| Diabetes mellitus | 465 (9.0) | 7,122 (14.3) | <0.001 |
| On antidiabetic treatment | 195 (3.8) | 1,542 (4.8) | <0.001 |
| High/low-density cholesterolemia | 229 (4.4) | 2,953 (6.0) | <0.001 |
| On lipid-lowering treatment | 79 (2.1) | 305 (1.4) | <0.001 |
| Mean characteristics |  |  |  |
| Age (year) | 46.9±9.6 | 48.9±13.9 | <0.001 |
| MAP (mm Hg) | 94.9±12.7 | 102.5±250.8 | 0.03 |
| Resting heart rate (bpm) | 72.3±10.8 | 74.7±11.2 | <0.001 |
| Waist circumference (cm) | 83.9±10.0 | 86.7±10.2 | <0.001 |
| BMI (kg/m^2^) | 24.2±2.8 | 25.0±3.5 | <0.001 |
| LDL- cholesterol (mmol/L) | 2.5±0.7 | 2.7±1.0 | <0.001 |
| FBG (mmol/L) | 5.4±1.4 | 5.8±2.2 | <0.001 |
| Uric acid (µmol/L) | 306.9±77.8 | 319.2±98.1 | <0.001 |
| eGFR (mL/min·1.73m^2^) | 100.2±19.2 | 97.8±23.4 | <0.001 |
| Hs-CRP (mg/L) | 1.08 (0.50,1.97) | 1.23 (0.62,2.31) | 0.03 |

BMI = body mass index; eGFR = estimated glomerular filtration rate; FBG = fasting blood glucose; Hs-CRP = hypersensitive C-reactive protein; LDL-C = low-density lipoprotein cholesterol; MAP = mean arterial pressure; RHR = resting heart rate. Reported values are number of participants (%), arithmetic means (±SD) or geometric means (interquartile range). Hypertension was an office blood pressure of ≥140 mmHg systolic or ≥90 mm Hg diastolic or use of antihypertensive drugs. Diabetes mellitus was a self-reported diagnosis, a fasting glucose level of ≥7.0mmol/L (126 mg/dL) or use of antidiabetic agents. The estimated glomerular filtration rate (eGFR) was calculated according to the Chronic Kidney Disease Epidemiology Collaboration formula.

**Supplementary Table 3.**  BaPWV by different trajectory groups in males.

|  | **Low** | **Medium-low** | **Medium-high** | **High** | **p value** |
| --- | --- | --- | --- | --- | --- |
| **Male (n=2,445)** |  |  |  |  |  |
| **baPWV1** | 12.8±1.2 | 15.2±1.5 | 17.5±2.0 | 20.8±2.1 | ＜0.001 |
| N1=2,441 | 870 | 993 | 446 | 132 |  |
| ≤14m/s | 755 (86.8) | 222 (22.4) | 10 (2.2) | 0 (0) | ＜0.001 |
| 14-18m/s | 111 (12.8) | 729 (73.4) | 273 (61.2) | 8 (6.1) |  |
| ＞18m/s | 4 (0.5) | 42 (4.2) | 163 (36.6) | 124 (94.0) |  |
| **baPWV2** | 13.0±1.3 | 15.6±1.4 | 18.4±1.9 | 21.1±2.0 | ＜0.001 |
| N2=2,438 | 868 | 992 | 445 | 133 |  |
| ≤14m/s | 696 (80.2) | 122 (12.3) | 6 (1.4) | 0 (0) | ＜0.001 |
| 14-18m/s | 170 (19.6) | 827 (83.4) | 181 (40.7) | 8 (6.0) |  |
| ＞18m/s | 2 (0.2) | 43 (4.3) | 258 (58.0) | 125 (94.0) |  |
| **baPWV3** | 13.2±1.3 | 15.8±1.5 | 18.7±2.1 | 21.2±2.0 | ＜0.001 |
| N3=2,435 | 870 | 990 | 446 | 129 |  |
| ≤14m/s | 640 (73.6) | 94 (9.5) | 8 (1.8) | 0 (0) | ＜0.001 |
| 14-18m/s | 229 (26.3) | 816 (82.4) | 151 (33.9) | 6 (4.7) |  |
| ＞18m/s | 1 (0.1) | 80 (8.1) | 287 (64.4) | 123 (95.4) |  |
| **baPWV4** | 13.4±1.6 | 16.1±1.6 | 19.1±2.4 | 21.3±2.3 | ＜0.001 |
| N4=637 | 233 | 255 | 113 | 35 |  |
| ≤14m/s | 147 (63.1) | 26 (10.2) | 2 (1.8) | 1 (2.9) | ＜0.001 |
| 14-18m/s | 86 (36.9) | 204 (80.0) | 35 (31.0) | 2 (5.7) |  |
| ＞18m/s | 0 (0) | 25 (9.8) | 76 (67.3) | 32 (91.4) |  |
| **baPWV annual growth rate (m/s/y)** | 0.08±0.37 | 0.13±0.55 | 0.29±0.82 | 0.06±0.81 | ＜0.001 |

baPWV = brachial-ankle pulse wave velocity.

**Supplementary Table 4.**  BaPWV by different trajectory groups in females.

|  | **Low** | **Medium-low** | **Medium-high** | **High** | **p value** |
| --- | --- | --- | --- | --- | --- |
| **Female (n=2,735)** |  |  |  |  |  |
| **baPWV1** | 11.4±1.1 | 13.55±1.26 | 16.2±1.7 | 19.9±2.2 | ＜0.001 |
| N1=2,735 | 1,364 | 866 | 385 | 120 |  |
| ≤14m/s | 1,343 (98.5) | 587 (67.8) | 27 (7.0) | 0 (0) | ＜0.001 |
| 14-18m/s | 21 (1.5) | 276 (31.9) | 295 (76.6) | 27 (22.5) |  |
| ＞18m/s | 0 (0) | 3 (0.4) | 63 (16.4) | 93 (77.5) |  |
| **baPWV2** | 11.8±1.1 | 14.0±1.4 | 16.8±1.9 | 20.5±2.3 | ＜0.001 |
| N2=2,715 | 1,364 | 864 | 373 | 115 |  |
| ≤14m/s | 1,335 (98.0) | 486 (56.3) | 16 (4.3) | 0 (0) | ＜0.001 |
| 14-18m/s | 28 (2.05) | 370 (42.8) | 278 (74.5) | 20 (2.9) |  |
| ＞18m/s | 0 (0) | 8 (0.9) | 79 (21.2) | 95 (82.6) |  |
| **baPWV3** | 11.9±1.1 | 14.4±1.4 | 17.7±2.0 | 20.6±2.3 | ＜0.001 |
| N3=2,727 | 1,364 | 865 | 382 | 116 |  |
| ≤14m/s | 1,337 (98.1) | 367 (42.4) | 6 (1.6) | 1 (0.9) | ＜0.001 |
| 14-18m/s | 26 (1.9) | 488 (56.4) | 218 (57.1) | 12 (10.3) |  |
| ＞18m/s | 0 (0) | 10 (1.2) | 158 (41.4) | 103 (88.8) |  |
| **baPWV4** | 12.0±1.2 | 14.6±1.3 | 17.4±1.9 | 21.1±2.3 | ＜0.001 |
| N4=1,205 | 649 | 377 | 135 | 44 |  |
| ≤14m/s | 622 (95.8) | 143 (37.9) | 4 (3.0) | 0 (0) | ＜0.001 |
| 14-18m/s | 27 (4.2) | 230 (61.0) | 85 (63.0) | 5 (11.4) |  |
| ＞18m/s | 0 (0) | 4 (1.1) | 46 (34.1) | 39 (88.6) |  |
| **baPWV annual growth rate (m/s/y)** | 0.1±0.3 | 0.2±0.4 | 0.3±0.5 | 0.1±0.6 | ＜0.001 |

baPWV = brachial-ankle pulse wave velocity.

**Supplementary Table 5. Fitting degree of the main risk factors for the trajectory models.**

|  |  | **Variable** | **-2 Log Likelihood** | **X^2^** | **p value** | **Nagelkerke pseudo-R^2^** | **Δpseudo-R^2^** |
| --- | --- | --- | --- | --- | --- | --- | --- |
| Model 1 | Step 0 | Intercept | 12,545.4 | - | - | 0 | 0 |
|  | Step 1 | MAP | 10,804.7 | 1,740.7 | <0.001 | 0.335 | 0.335 |
|  | Step 2 | Age | 9,450.2 | 1,354.5 | <0.001 | 0.499 | 0.164 |
|  | Step 3 | Resting heart rate | 9,338.4 | 111.8 | <0.001 | 0.512 | 0.013 |
|  | Step 4 | Gender | 9,218.4 | 120.0 | <0.001 | 0.525 | 0.013 |
|  | Step 5 | FBG | 9,137.4 | 81.0 | <0.001 | 0.534 | 0.009 |
|  | Step 6 | Uric acid | 9,121.5 | 15.9 | 0.001 | 0.536 | 0.002 |
|  | Step 7 | Drinking alcohol | 9,109.9 | 11.6 | 0.009 | 0.537 | 0.001 |
|  | Step 8 | Hs-CRP | 9,098.7 | 11.1 | 0.01 | 0.539 | 0.002 |
|  | Step 9 | LDL-cholesterol | 9,088.8 | 10.0 | 0.02 | 0.540 | 0.001 |
|  |  |  |  |  |  |  |  |
| Model 2 | Step 0 | Intercept | 12,545.4 | - | - | 0 | 0 |
|  | Step 1 | MAP | 10,804.7 | 1,740.7 | <0.001 | 0.335 | 0.335 |
|  | Step 2 | Age | 9,450.2 | 1,354.5 | <0.001 | 0.499 | 0.164 |
|  | Step 3 | Δ MAP | 8,961.7 | 488.6 | <0.001 | 0.553 | 0.054 |
|  | Step 4 | Resting heart rate | 8,854.0 | 107.7 | <0.001 | 0.565 | 0.012 |
|  | Step 5 | Gender | 8,779.9 | 74.1 | <0.001 | 0.572 | 0.007 |
|  | Step 6 | FBG | 8,720.8 | 59.1 | <0.001 | 0.578 | 0.006 |
|  | Step 7 | Δ Resting heart rate | 8,689.3 | 31.5 | <0.001 | 0.582 | 0.004 |
|  | Step 8 | Δ FBG | 8,670.1 | 19.2 | <0.001 | 0.584 | 0.002 |
|  | Step 9 | LDL-cholesterol | 8,656.7 | 13.5 | 0.004 | 0.585 | 0.001 |
|  | Step 10 | Δ LDL-cholesterol | 8,644.3 | 12.4 | 0.006 | 0.586 | 0.001 |
|  | Step 11 | Δ Uric acid | 8,633.0 | 11.2 | 0.01 | 0.587 | 0.001 |
|  | Step 12 | Uric acid | 8,621.4 | 11.6 | 0.009 | 0.588 | 0.001 |
|  | Step 13 | BMI | 8,610.1 | 11.3 | 0.01 | 0.589 | 0.001 |
|  | Step 14 | Drinking alcohol | 8,598.8 | 11.4 | 0.01 | 0.591 | 0.002 |

BMI = body mass index; FBG = fasting blood glucose; Hs-CRP = hypersensitive C-reactive protein; LDL = low-density lipoprotein; MAP = mean arterial pressure. Δ was the difference of last follow-up data and baseline data. Gender (female) is the reference group. Quasi-natural ratio test is used to test the fitting degree of the model by adding independent variables of multiple logistic regression analysis through the forward stepwise method (enter probability: 0.05, remove probability: 0.10), and the change value of Nagelkerke pseudo-R^2^ is used to evaluate the explanation of the added independent variable to the dependent variable variation degree. Model 2: based on Model 1, Δ MAP, Δ Resting heart rate, Δ FBG, Δ LDL- cholesterol, and Δ Uric acid are additionally adjusted.

**Supplementary Table 6. Sensitive analyses by excluding participants with antihypertensive treatment or incidence of hypertension, diabetes mellitus, or cardiovascular disease.**

**Suppl Table 6a. Sensitive analyses by excluding participant with antihypertensive treatment.**

|  |  | **Low (n=1,961)** | **Medium-low (n=1,846)** | **Medium-high (n=1,024)** | **High (n=351)** |
| --- | --- | --- | --- | --- | --- |
| Sensitivity 1  (n=4,409) | Age | Ref. | 1.08 (1.07-1.09) | 1.20 (1.18-1.22) | 1.28 (1.24-1.31) |
|  | Male | Ref. | 1.80 (1.49-2.19) | 2.15 (1.63-2.82) | 3.08 (1.94-4.88) |
|  | MAP | Ref. | 1.11 (1.10-1.12) | 1.20 (1.18-1.22) | 1.27 (1.24-1.30) |
|  | Resting heart rate | Ref. | 1.03 (1.02-1.04) | 1.06 (1.05-1.07) | 1.08 (1.06-1.11) |
|  | BMI | Ref. | 0.95 (0.92-0.98) | 0.95 (0.90-0.99) | 0.85 (0.79-0.92) |
|  | FBG | Ref. | 1.30 (1.17-1.45) | 1.48 (1.31-1.67) | 1.65 (1.43-1.91) |
|  | LDL- cholesterol | Ref. | 1.25 (1.11-1.41) | 1.36 (1.15-1.62) | 1.10 (0.84-1.45) |
|  | Uric acid | Ref. | 1.002 (1.000-1.003) | 1.004 (1.002-1.006) | 1.001 (0.998-1.004) |
|  | Δ MAP | Ref. | 1.06 (1.05-1.07) | 1.11 (1.09-1.12) | 1.14 (1.12-1.16) |
|  | Δ Resting heart rate | Ref. | 1.01 (1.00-1.02) | 1.02 (1.01-1.03) | 1.02 (1.00-1.03) |
|  | Δ FBG | Ref. | 1.16 (1.06-1.28) | 1.20 (1.08-1.34) | 1.34 (1.18-1.52) |
|  | Δ Uric acid | Ref. | 1.002 (1.000-1.003) | 1.003 (1.001-1.005) | 1.004 (1.002-1.007) |

BMI = body mass index; FBG = fasting blood glucose; Hs-CRP = hypersensitive C-reactive protein; LDL = low-density lipoprotein; MAP = mean arterial pressure. Δ was the difference of last follow-up data and baseline data. Reported values are odds ratios with 95% confidence intervals. Sensitivity 1: Participants taking antihypertensive medications are excluded, Nagelkerke pseudo-R^2^=0.557.

**Suppl Table 6b. Sensitive analyses by excluding participant with incidence of hypertension.**

|  |  | **Low (n=1,961)** | **Medium-low (n=1,846)** | **Medium-high (n=1,024)** | **High (n=351)** |
| --- | --- | --- | --- | --- | --- |
| Sensitivity 2  (n=4,612) | Age | Ref. | 1.08 (1.07-1.10) | 1.20 (1.18-1.22) | 1.29 (1.26-1.31) |
|  | Male | Ref. | 1.77 (1.45-2.18) | 1.82 (1.39-2.38) | 1.88 (1.28-2.74) |
|  | MAP | Ref. | 1.11 (1.10-1.12) | 1.20 (1.18-1.22) | 1.24 (1.22-1.26) |
|  | Resting heart rate | Ref. | 1.02 (1.01-1.03) | 1.05 (1.04-1.07) | 1.07 (1.05-1.09) |
|  | BMI | Ref. | 0.97 (0.94-1.01) | 0.96 (0.91-1.01) | 0.91 (0.85-0.97) |
|  | FBG | Ref. | 1.26 (1.13-1.40) | 1.40 (1.24-1.57) | 1.63 (1.43-1.85) |
|  | LDL-cholesterol | Ref. | 1.25 (1.09-1.43) | 1.21 (1.01-1.46) | 1.09 (0.85-1.39) |
|  | Hs-CRP | Ref. | 1.03 (1.00-1.07) | 1.08 (1.04-1.13) | 1.06 (1.00-1.12) |
|  | Uric acid | Ref. | 1.002 (1.000-1.004) | 1.003 (1.001-1.005) | 1.004 (1.001-1.006) |
|  | Drinking alcohol | Ref. | 0.81 (0.54-1.20) | 1.19 (0.77-1.86) | 1.72 (1.00-2.94) |
|  | Δ MAP | Ref. | 1.05 (1.04-1.06) | 1.10 (1.09-1.12) | 1.12 (1.10-1.14) |
|  | Δ Rest heart rate | Ref. | 1.01 (1.00-1.02) | 1.02 (1.01-1.03) | 1.01 (1.00-1.02) |
|  | Δ FBG | Ref. | 1.12 (1.02-1.23) | 1.21 (1.09-1.34) | 1.27 (1.13-1.43) |
|  | Δ LDL-cholesterol | Ref. | 1.09 (0.97-1.23) | 0.95 (0.81-1.12) | 0.84 (1.68-1.05) |
|  | Δ Hs-CRP | Ref. | 1.03 (1.00-1.06) | 1.05 (1.01-1.08) | 1.04 (1.00-1.09) |
|  | Δ Uric acid | Ref. | 1.002 (1.000-1.003) | 1.003 (1.001-1.005) | 1.005 (1.003-1.008) |

BMI = body mass index; FBG = fasting blood glucose; Hs-CRP = hypersensitive C-reactive protein; LDL = low-density lipoprotein; MAP = mean arterial pressure. Δ was the difference of last follow-up data and baseline data. Reported values are odds ratios with 95% confidence intervals. Sensitivity 2: Participants with new-onset hypertension during the baPWV follow-up are excluded, Nagelkerke pseudo-R^2^=0.603.

**Suppl Table 6c. Sensitive analyses by excluding participant with incidence of diabetes mellitus.**

|  |  | **Low (n=1,961)** | **Medium-low (n=1,846)** | **Medium-high (n=1,024)** | **High (n=351)** |
| --- | --- | --- | --- | --- | --- |
| Sensitivity 3  (n=4,973) | Age | Ref. | 1.08 (1.07-1.10) | 1.20 (1.18-1.22) | 1.30 (1.27-1.32) |
|  | Male | Ref. | 1.55 (1.24-1.94) | 1.51 (1.13-2.02) | 1.47 (0.97-2.22) |
|  | MAP | Ref. | 1.11 (1.10-1.12) | 1.20 (1.18-1.22) | 1.25 (1.23-1.27) |
|  | Resting heart rate | Ref. | 1.03 (1.02-1.04) | 1.06 (1.05-1.07) | 1.08 (1.06-1.09) |
|  | FBG | Ref. | 1.20 (1.09-1.33) | 1.34 (1.20-1.49) | 1.56 (1.38-1.75) |
|  | LDL- cholesterol | Ref. | 1.25 (1.10-1.43) | 1.24 (1.04-1.47) | 1.05 (0.83-1.35) |
|  | Uric acid | Ref. | 1.002 (1.000-1.003) | 1.004 (1.002-1.005) | 1.003 (1.001-1.006) |
|  | Current smoking | Ref. | 1.40 (1.09-1.81) | 1.48 (1.09-2.01) | 1.48 (0.97-2.24) |
|  | Drinking alcohol | Ref. | 0.76 (0.52-1.11) | 1.04 (0.68-1.60) | 1.65 (0.96-2.83) |
|  | Δ MAP | Ref. | 1.06 (1.05-1.07) | 1.11 (1.09-1.12) | 1.13 (1.11-1.15) |
|  | Δ Resting heart rate | Ref. | 1.01 (1.00-1.02) | 1.02 (1.01-1.03) | 1.01 (1.00-1.02) |
|  | Δ FBG | Ref. | 1.06 (0.96-1.17) | 1.13 (1.01-1.26) | 1.18 (1.04-1.34) |
|  | Δ LDL- cholesterol | Ref. | 1.11 (0.99-1.24) | 0.94 (0.80-1.09) | 0.80 (0.65-1.00) |
|  | Δ Uric acid | Ref. | 1.001 (1.000-1.003) | 1.003 (1.001-1.004) | 1.005 (1.002-1.007) |

BMI = body mass index; FBG = fasting blood glucose; Hs-CRP = hypersensitive C-reactive protein; LDL = low-density lipoprotein; MAP = mean arterial pressure. Δ was the difference of last follow-up data and baseline data. Reported values are odds ratios with 95% confidence intervals. Sensitivity 3: Participants with new-onset diabetes during the baPWV follow-up are excluded, Nagelkerke's pseudo-R2=0.592.

**Suppl Table 6d. Sensitive analyses by excluding participant with incidence of cardiovascular disease.**

|  |  | **Low (n=1,961)** | **Medium-low (n=1,846)** | **Medium-high (n=1,024)** | **High (n=351)** |
| --- | --- | --- | --- | --- | --- |
| Sensitivity 4  (n=5,112) | Age | Ref. | 1.08 (1.07-1.09) | 1.20 (1.18-1.22) | 1.29 (1.27-1.32) |
|  | Male | Ref. | 1.80 (1.49-2.19) | 1.84 (1.43-2.37) | 1.91 (1.32-2.76) |
|  | MAP | Ref. | 1.11 (1.10-1.12) | 1.20 (1.18-1.22) | 1.25 (1.23-1.27) |
|  | Resting heart rate | Ref. | 0.96 (0.93-0.99) | 1.06 (1.05-1.07) | 1.08 (1.06-1.09) |
|  | BMI | Ref. | 1.24 (1.12-1.36) | 0.95 (0.91-0.99) | 0.90 (0.85-0.96) |
|  | FBG | Ref. | 1.27 (1.12-1.45) | 1.37 (1.23-1.53) | 1.62 (1.44-1.83) |
|  | LDL- cholesterol | Ref. | 1.27 (1.12-1.36) | 1.23 (1.03-1.45) | 1.10 (0.86-1.40) |
|  | Hs-CRP | Ref. | 1.02 (0.99-1.04) | 1.04 (1.01-1.08) | 1.01 (0.96-1.06) |
|  | Uric acid | Ref. | 1.002 (1.000-1.003) | 1.003 (1.001-1.005) | 1.003 (1.001-1.006) |
|  | Drinking alcohol | Ref. | 0.80 (0.56-1.15) | 1.10 (0.73-1.66) | 1.54 (0.92-2.60) |
|  | Δ MAP | Ref. | 1.06 (1.05-1.07) | 1.11 (1.09-1.12) | 1.13 (1.11-1.15) |
|  | Δ Resting heart rate | Ref. | 1.01 (1.00-1.02) | 1.02 (1.01-1.03) | 1.01 (1.00-1.02) |
|  | Δ FBG | Ref. | 1.13 (1.03-1.23) | 1.20 (1.09-1.32) | 1.30 (1.16-1.45) |
|  | Δ LDL- cholesterol | Ref. | 1.08 (0.97-1.21) | 0.91 (0.79-1.06) | 0.84 (0.68-1.03) |
|  | Δ Uric acid | Ref. | 1.001 (1.000-1.003) | 1.003 (1.001-1.004) | 1.005 (1.003-1.007) |

BMI = body mass index; FBG = fasting blood glucose; Hs-CRP = hypersensitive C-reactive protein; LDL = low-density lipoprotein; MAP = mean arterial pressure. Δ was the difference of last follow-up data and baseline data. Reported values are odds ratios with 95% confidence intervals. Sensitivity 4: Participants with new-onset CVD during the baPWV follow-up are excluded, Nagelkerke's pseudo-R^2^=0.588.
